# Dengue prevention: A community-based study on knowledge and practices

**DOI:** 10.6026/973206300223197

**Published:** 2026-05-31

**Authors:** Alka Modi Asat, Amrutha R, Sunita Vishwakarma, Sunil Kant Guleri, Soumitra Sethia, Sachin Gupta, Konika Jain, Durgesh Shukla, Rohit Das

**Affiliations:** 1 Department of Community Medicine, Sunderlal Patwa Government Medical College, Mandsaur, Madhya Pradesh, India

**Keywords:** Dengue, Aedes aegypti, larval indices, health promotion

## Abstract

Despite dengue being a major public health threat in urban India, there is limited local evidence on household larval indices and community knowledge and practices, particularly in smaller urban settings. Hence, this cross-sectional study (July-September 2025) assessed knowledge, practices and larval indices across 1061 households. Moderate knowledge existed, with good practices and larvae were found in 195 homes (House Index 18.4%). Waterlogging (OR=17.37), low SES (OR=1.62) and poor storage (OR=2.56) predicted larvae (all p<0.001). Thus, post-survey health education was implemented. Therefore, targeted vector control is recommended.

## Background:

Vector-borne diseases account for more than 17% of all infectious diseases and remain a major public health challenge in India and globally [1]. Dengue threatens 3.9 billion people globally with 96 million cases and 40,000 deaths annually; 2024 saw record highs of 14 million cases and 11,000 deaths. WHO declared it a Grade-3 emergency in 2023 [2, 3]. Transmission peaks during and after the rainy season, influenced by temperature, rainfall, humidity, mosquito density and community behaviors [4]. In South Delhi, gaps persisted in identifying Aedes mosquitoes and recognizing breeding sites despite high general awareness [5], while targeted health education in Tamil Nadu significantly improved KAP [6]. Community-based interventions in Indonesia demonstrated reduction in larval indices following structured health promotion [7]. However, limited literature exists on local larval prevalence and community practices in Mandsaur city, this study aims to assess household larval indices and evaluate baseline community knowledge and preventive practices among urban residents during the monsoon season. Therefore, it is of interest to identify socio-demographic determinants influencing preventive behaviors and to implement targeted health promotional activities designed to strengthen local dengue prevention strategies.

## Methodology:

This community-based cross-sectional study was conducted during July-September 2025 across seven high-risk areas of Mandsaur city, identified by the District Malaria Centre under NVBDCP. A total of 1061 households were selected through systematic random sampling (high-risk wards → streets/blocks → alternate households), with one consenting adult per household surveyed using a pre-tested Hindi questionnaire covering socio-demographics, dengue knowledge and preventive practices. The sample size was calculated using the formula, n=Z^2^pq/d^2^(Z=1.96, d=5%, assumed dengue knowledge prevalence=50%), adjusted with a design effect of 2.5 and 10% non-response rate to yield 1061 participants. Inclusion criteria comprised households with residents living ≥6 months and willing to consent; exclusions were vacant houses or absent adults. Household larval surveys (Figure 1 see PDF) followed WHO guidelines, inspecting indoor/outdoor water containers to identify Aedes larvae and calculate larval indices (House Index, Container Index, Breteau Index). Post- survey, health promotion included one-to-one counseling (emphasizing larva-positive and low-SES homes), live demonstrations on source reduction (emptying/cleaning/covering containers, cooler management, eliminating stagnant water) and guidance on environmental sanitation/ community clean-up drives (Figure 1 see PDF). Outcome variables were knowledge/practice scores, larval presence and indices. Data were entered in Microsoft Excel and analyzed using Epi Info/Jamovi (means ±SD, Pearson correlation for knowledge-practice; p<0.05 significant; r -1 to 0 negative, 0 to 1 positive correlation).

## Ethical consideration:

As this activity was undertaken as part of routine surveillance and field operations under the National Vector Borne Disease Control Program (NVBDCP) in Mandsaur district, Madhya Pradesh, appropriate programmatic and administrative approvals were obtained from the competent district health authorities for documentation and publication of the study. Figure 1 (see PDF) showing Health promotional activity related mosquito borne disease, larva survey in community, destruction of larva.

## Results and Discussion:

A total of 1,061 participants were included in the study. Mosquito larvae were detected in 195 households. Over half of the participants (51.8%) were aged 20-40 years and 80.7% were males. Most participants had primary education (39.8%) or no formal schooling (29.9%). Nearly half (45.4%) were skilled workers, while 29.2% were unskilled and 18.0% unemployed. The majority (67.4%) belonged to the upper-lower socio-economic class. About 15% of households had set-back areas, 18.3% reported water stagnation nearby and 7.0% kept pets or cattle within their premises. Among 1,061 participants, knowledge about dengue transmission was moderate, with a mean score of 4.0. Strengths included 69.5% (737) correctly identifying the best prevention as using mosquito nets and eliminating breeding sites, 62.5% (663) recognizing government strategies like spraying insecticides and fogging and 53.4% (567) knowing dengue is curable. However, gaps persisted in vector biology: only 58.4% (620) knew transmission occurs via the bite of an infected female Aedes mosquito, 47.6% (505) understood people can get dengue more than once, just 28.7% (305) recognized the mosquito's most active periods as early morning and late afternoon and 39.8% (422) identified clean stagnant water in containers as breeding sites. Knowledge regarding common symptoms of dengue among participants is illustrated in Figure 2 (see PDF). Similar findings were reported in Rohtak, found relatively high awareness of symptoms and prevention, yet poor understanding of biting time and breeding places of Aedes mosquitoes, especially among less-educated groups [8]. Similar to studies in Pune, where irregular water supply and environmental conditions contributed to breeding [9]. The preventive practices adopted by study participants are summarized in Table 1. Preventive practices were generally good, with a mean score of 6.1, especially in personal protection and water container management, although timely healthcare seeking remain very low. Similar to studies in Maharashtra, where despite increasing awareness, dengue continues to remain a significant public health problem due to gaps in effective implementation of preventive practices [10]. However, the persistent presence of breeding sites suggests that practices may not be consistently or adequately implemented. This study showed a moderate level of knowledge and a relatively higher and more consistent level of practice among participants. The higher standard deviation in knowledge scores indicates greater variability in knowledge levels, whereas practices were more uniformly adopted. Comparable studies in Aligarh, where knowledge about vector-borne diseases varies widely across communities, but practice levels do not always align perfectly with knowledge [11]. Knowledge-practice disparity was evident in the present study. Although 96.6% of participants reported using personal protection, only 28.7% knew that Aedes mosquitoes bite during the daytime, which may influence protective behavior. Similar study conducted among 1,016 individuals, where important deficiencies in dengue-related knowledge and preventive practices persisted despite reasonable awareness [16]. Dengue larvae presence strongly associated with key factors. Houses with water logging showed a markedly higher presence of dengue larvae (61.6% vs. 8.4%), with a highly significant association (p < 0.001; OR = 17.37) and larvae were more common in lower socioeconomic groups (19.9% vs. 13.3%; p < 0.001; OR = 1.62). Households with poor practices had more than double the proportion of dengue larvae (28.4% vs. 13.4%), demonstrating a strong and significant association between inadequate preventive practices and larval presence (p < 0.001; OR = 2.56). similar to studies in Gautam Buddha Nagar [12]. Poor drainage, irregular water supply and inadequate waste management are likely to contribute to higher breeding. This aligns with recent findings from Uttar Pradesh highlighting that rapid urbanization combined with poor environmental sanitation severely exacerbates dengue outbreaks, necessitating active disease surveillance and strict containment measures in vulnerable communities [14]. The correlation matrix showing relationships between knowledge, practice and socio-demographic variables is presented in Table 2. Higher knowledge was associated with better education (r = 0.136), occupation status (r = 0.068) and socio-economic class (r = 0.122) all statistically significant (p < 0.001) indicating that more educated and better-off individuals had slightly better dengue knowledge; knowledge also showed a meaningful positive link with good practices (r = 0.336, p < 0.001), while increasing age was linked to lower knowledge (r = -0.157, p < 0.001); practice scores themselves were significantly influenced only by knowledge, not by socio-demographic factors. Mosquito larval indices were House Index 18.4%, Container Index 13.8% and Breteau Index 49%, indicating ongoing transmission risk consistent with finding from central India and Delhi where vector related knowledge gaps persisted despite awareness [13]. Recent entomological studies in Central India reinforce these risks, demonstrating the growing dominance and co-circulation of both Aedes aegypti and Aedes albopictus in urban environments, heavily driven by stagnant water in domestic containers [15]. Post-survey health promotion addressed immediate gaps, but sustained community engagement is needed for long-term behavior change.

## Conclusion:

We report that although general awareness about dengue exists, critical gaps remain in understanding mosquito behaviour and reinfection risk. Preventive practices were relatively good, yet environmental sanitation issues, especially waterlogging, continue to facilitate breeding. Lower socio-economic households were disproportionately affected. Strengthened health education, environmental management and community participation are essential for effective dengue control in Mandsaur city.

## Recommendations:

Strengthening IEC/BCC campaigns is important to improve awareness about mosquito biting time, reinfection risk and weekly dry-day practices. Household preventive measures should be encouraged, such as weekly cleaning of coolers, water tanks and containers. Environmental management should be improved through better drainage, proper waste management and surveillance of mosquito breeding hotspots. Government vector control activities should be strengthened with regular inspections, fogging and adequate larvicide supply. Community participation should be promoted through clean-up drives and ward-level dengue prevention committees. Special focus should be given to vulnerable groups, particularly lower socioeconomic households with higher breeding risk. Regular evaluation using larval indices and periodic KAP assessments should be conducted to monitor the effectiveness of dengue prevention efforts.

## Advancement of knowledge:

This study advances dengue epidemiology by revealing a critical disconnect between high self-reported preventive practices (96.6%) and actual environmental risks in Mandsaur. It reports that waterlogging (OR=17.37) and poor water storage (OR=2.56) strongly predict household larval presence (House Index 18.4%), despite moderate community awareness. By linking socio-demographic factors directly with larval indices, this study highlights the urgent need to shift from purely educational interventions toward structural environmental management in lower socio-economic areas.

## Limitations:

[1] Cross-sectional design limits temporal or causal interpretations.

[2] Self-reported practices may involve recall or social desirability bias.

[3] Study limited to selected high-risk areas; not generalizable to all urban settings.

[4] Observer variability in larval detection may occur.

[5] Slight gender imbalance in respondents.

## Figures and Tables

**Table 1:** Practices of participants to prevent dengue (correct responses).

**Good Practice**	**Frequency, n (%)**
Water containers to be emptied and cleaned	877 (82.7%)
The family regularly covers water storage containers.	799 (75.3%)
Clean overhead tanks / coolers	873 (82.3%)
Eliminate stagnant water around the house	686 (64.7%)
Use of personal protection against mosquitoes	1025 (96.6%)
Allows door-to-door government spraying or not.	737 (69.5%)
Participated in community clean-up drives or OPD camps related to dengue.	546 (51.5%)
Seek healthcare for fever	856 (80.7%)
When do you seek healthcare for fever (Within 24 hours of onset of symptoms)	39 (3.6%)

**Table 2:** Correlation matrix of knowledge, practice and socio-demographic variable

		**Total K Score**	**Education Score**	**Occupation score**	**SE Score**	**Age of respondent (In years)**	**Total P Score**
**Total K Score**	**Pearson's r**	-					
	df	-					
	p-value	-					
Education Score	Pearson's r	0.136	-				
	df	1059	-				
	p-value	<.001	-				
Occupation-score	Pearson's r	0.068	0.355	-			
	df	1059	1059	-			
	p-value	0.026	<.001	-			
SE Score	Pearson's r	0.122	0.706	0.862	-		
	df	1059	1059	1059	-		
	p-value	<.001	<.001	<.001	-		
Age of respondent (In years)	Pearson's r	-0.157	-0.235	-0.014	-0.097	-	
	df	1059	1059	1059	1059	-	
	p-value	<.001	<.001	0.639	0.002	-	
Total P Score	Pearson's r	0.336	-0.017	0.02	-0.02	-0.004	-
	df	1059	1059	1059	1059	1059	-
	p-value	<.001	0.572	0.519	0.511	0.896	-
